# Subwavelength nonlinear phase control and anomalous phase matching in plasmonic metasurfaces

**DOI:** 10.1038/ncomms10367

**Published:** 2016-01-22

**Authors:** Euclides Almeida, Guy Shalem, Yehiam Prior

**Affiliations:** 1 Department of Chemical Physics, Weizmann Institute of Science, Rehovot 76100, Israel

## Abstract

Metasurfaces, and in particular those containing plasmonic-based metallic elements, constitute an attractive set of materials with a potential for replacing standard bulky optical elements. In recent years, increasing attention has been focused on their nonlinear optical properties, particularly in the context of second and third harmonic generation and beam steering by phase gratings. Here, we harness the full phase control enabled by subwavelength plasmonic elements to demonstrate a unique metasurface phase matching that is required for efficient nonlinear processes. We discuss the difference between scattering by a grating and by subwavelength phase-gradient elements. We show that for such interfaces an anomalous phase-matching condition prevails, which is the nonlinear analogue of the generalized Snell's law. The subwavelength phase control of optical nonlinearities paves the way for the design of ultrathin, flat nonlinear optical elements. We demonstrate nonlinear metasurface lenses, which act both as generators and as manipulators of the frequency-converted signal.

## Abstract (web summary)

The fundamental issue of phase-matching across metasurfaces has not been thoroughly addressed. Here, Almeida *et al*. show full phase control by introducing a spatially varying phase response of a metallic metasurface consisting of subwavelength nanoantennas and demonstrate metasurface-phase-matching.

## Graphical abstract

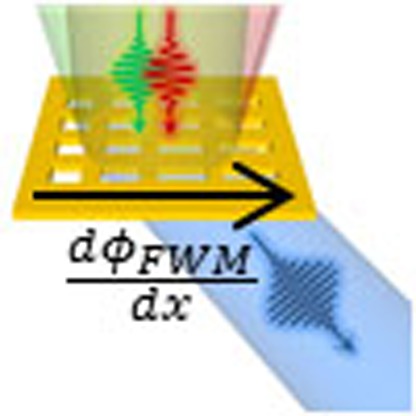

Metamaterials are a class of artificial materials whose optical properties can be tailored to exhibit phenomena not commonly found in nature, such as negative^1^,^2^ or anomalous refraction^3^,^4^. These unique optical properties are frequently engineered by single- or multi-layered nanometric objects, often metallic, fabricated on the surface of ‘classical' standard materials. Metasurfaces constitute a particularly interesting and attractive subset of such materials leading to the possibility of designing and creating, by means of modern nanolithographic fabrication techniques, flat and ultrathin optical elements^4^,^5^,^6^,^7^,^8^. Nano-plasmonic-based metallic elements are the commonly utilized building blocks, and their (linear) optical properties are quite well understood^6^,^7^. The significance of phase changes across a metasurface has been recognized early on^9^ and the laws of refraction across such surfaces have been recently reformulated by Yu *et al*.^4^ in terms of a generalized Snell's law. For metasurfaces with linear phase gradient^5^,^6^,^7^, it was shown that the transverse phase gradient d*Φ*/d*x* must be accounted for as light crosses the metasurface (ref. 4, equation (2)). Based on these principles, the same group^10^ demonstrated achromatic lenses by proper design of the phase elements in metasurfaces.

Metal-based metasurfaces can enhance optical nonlinearities at plasmonic resonances by orders of magnitude^11^. Negative refraction^12^ and zero index materials^13^ were demonstrated, enhancement in clusters and Fano resonances^14^ were shown, and the potential for super resolution in plasmonically enhanced four-wave mixing (FWM) was also discussed^15^. As for nonlinear harmonic generation, second harmonic generation (SHG) was studied^16^,^17^,^18^ and recently Segal *et al*.^19^ and Li *et al*.^20^ discussed the SHG signal generated on metasurfaces and demonstrated beam bending and focusing of such light. Other works, Lee *et al*.^21^ and Wolf *et al*.^22^, reported the use of metasurfaces to gain control over a SHG signal generated within the nonlinear substrate on which the metasurface is located. The next, third order, nonlinearity was investigated even less, although, in principle, it exists for any structure and for any surface symmetry. As an example, FWM was demonstrated for cavities perforated on gold films^23^. However, to the best of our knowledge, the fundamental issue of phase matching across metasurfaces has not yet been thoroughly addressed. This is, in part, due to the somewhat limited phase and amplitude control over the nonlinearities of individual plasmonic element, which in many cases drives researchers to resort to periodic structures (gratings) that impose specific angles of diffraction^24^.

Based on the linear results, one may anticipate that transverse phase gradients at the interface provide an additional momentum that must be included in any nonlinear phase-matching scheme. Here, we demonstrate full phase control over nonlinear optical interactions in plasmonic metasurfaces. This control is achieved by introducing a spatially varying phase response of a metallic metasurface consisting of subwavelength nonlinear nanoantennas designed specifically for the frequency of the nonlinear signal. For such metasurfaces, we derive a new, anomalous nonlinear phase-matching condition that differs from the conventional phase-matching schemes in nonlinear optics. The complete phase control over the nonlinear emission enables us to design flat nonlinear optical components, such as nonlinearly blazed elements and ultrathin frequency-converting lenses with tight focusing.

## Results

### Finite-differences time-domain calculations

A coherent wave-mixing process obeys the phase-matching condition Δ**k**=0, where Δ**k** is the vectorial sum of the momenta of all photons, incoming and generated, participating in the nonlinear mixing. This condition determines the direction of the coherent emission. In FWM, for collinear input beams in a homogeneous and non-dispersive medium, the phase-matched generated beam propagates in the same direction, and this is also the case for the quasi-phase-matching scheme^25^,^26^, where a nonlinear material is designed with periodic reversal of the sign of the nonlinearity in the propagation direction. For non-collinear input beams, a more elaborate phase-matching scheme is required, often based on birefringence or temperature tuning. For our metasurfaces, the nonlinear phase gradient imposed by the plasmonic antennas determines the phase-matching conditions.

Optical nanoantennas, like other driven oscillators, reradiate the incoming light at the same frequency but with a shifted phase. In this work, we use rectangular nanocavities in thin gold films as optical antennas, and to convey the new physical principles more clearly, the aspect ratio (AR) of the rectangles was maintained as our single tuning parameter.

**Figure 1:**
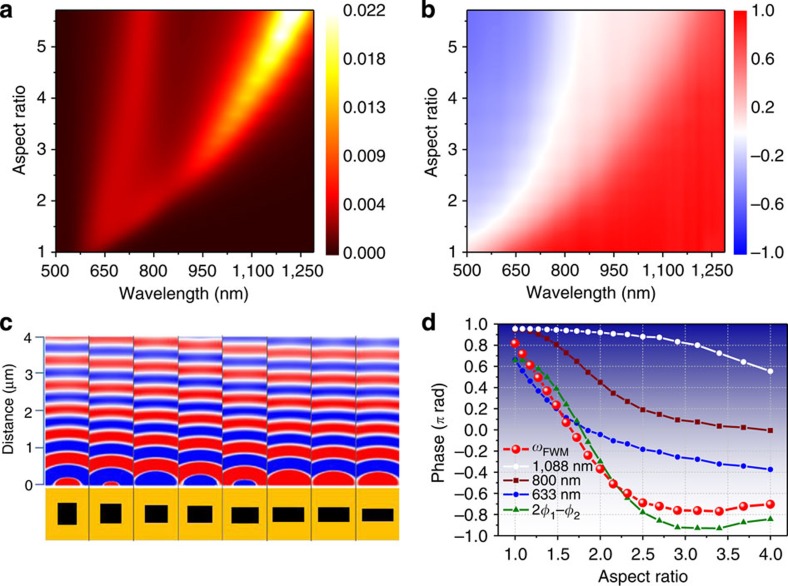
Linear and FWM transmission, through rectangular gold nanocavities with varying aspect ratios. (**a**) Linear transmittance spectral intensity and (**b**) corresponding linear spectral phase (colour code is in units of *π*) (**c**) The calculated phase of the generated FWM field *E*_*y*_(*ω*_FWM_)/|*E*_*y*_(*ω*_FWM_)| at the exit from the film as it propagates away from the surface. (**d**) The phases accumulated by the different fields (input and generated) on crossing the metasurface as a function of the aspect ratio: 1,088 nm—white circles, 800 nm—brown squares, 633 nm (independently propagating)—blue circles, generated FWM at 633 nm—red circles and the phase accumulated at 2*Φ*_1_−*Φ*_2_—green triangle (see text).

In Fig. 1, we present nonlinear finite-differences time-domain (FDTD) calculations (details given in the Methods section) performed for individual cavities of varying ARs. These separate calculations are combined to depict the transmission and nonlinear interactions in phased arrays of such cavities. In Fig. 1a,b, we show the calculated linear transmittance spectrum (intensity and phase) for light polarized along the short axis of the rectangles for a set of rectangular nanocavities of different ARs within a free-standing 250-nm thick gold film. Two distinct cavity resonances are seen^27^, and the correlation between the intensity and the phase shift acquired by the transmitted wave is clear.

Consider now a FWM configuration where two transform-limited laser pulses (with *ω*_*j*_, **k**_*j*_ and 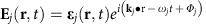 where *j*=1, 2, respectively) interact with a metallic nanoantenna to generate a FWM signal 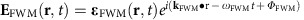 travelling at the **k**_FWM_ direction and with frequency *ω*_FWM_=2*ω*_1_−*ω*_2_. The third-order polarization induced at position **r** is given by^28^:


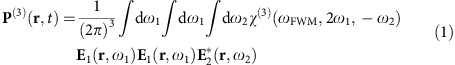


Where *χ*^(3)^ is the third-order susceptibility of the metal and the fields **E**_*i*_(**r**,*ω*_*i*_) are the position-dependent electric fields. An antenna much smaller than the wavelength can be approximated by a point dipole (eliminating the position dependence within the antenna) and this leads to effective fields^29^, 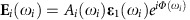 where *A*_*i*_(*ω*_*i*_) is the field enhancement (a real quantity) and *Φ*(*ω*_*i*_) is the phase response. Therefore, we can rewrite equation (1) as


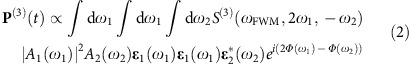


Where *S*^(3)^ is the effective third-order nonlinear susceptibility. The nonlinear FWM signal carries the frequency response at the fundamental frequencies through the phase factor 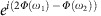, which changes sharply for excitation close to the nanoantenna resonance. The nonlinear phase response of the nanoantennas can be directly calculated using full-wave nonlinear finite-differences time-domain (NL-FDTD) calculations.

In Fig. 1c,d, we show the generated field at *ω*_FWM_ for a set of nanorectangles with varying AR. In the NL-FDTD calculation, two 60-fs long transformed-limited co-propagating pulses, with centre frequencies *ω*_1_=800 nm and *ω*_2_=1,088 nm, respectively, are temporally and spatially overlapped at a single nanocavity. Figure 1d depicts the linear phase accumulated by individually propagating waves at 800, 1,088 and 633 nm, and the nonlinear phase accumulated by the generated FWM beam at 633 nm. The calculated *Φ*_FWM_ does not fit the phase of the 633-nm wave. The calculated phase difference, 2*Φ*_1_−*Φ*_2_, provides a much better estimate for the phase of the generated FWM signal, but the fit is still not perfect.

For a better description of the generated phase two additional factors need be included. The first, and less critical one, is integration over the laser bandwidth, which for these ultrashort pulses is not negligible. The more important factor is the fact that the FWM signal is generated gradually over the length of propagation through the metasurface, so that the phase accumulated by this wave as it is building up should also be included, and can be incorporated as an effective dielectric constant 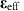 that appears in the nonlinear wave equation


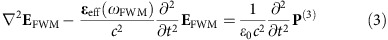


As mentioned, our direct NL-FDTD calculation provides the phase *Φ*_FWM_, and therefore we will use results such as of Fig. 1d for the design of our metasurface.

### Experimental arrangement and observations

**Figure 2:**
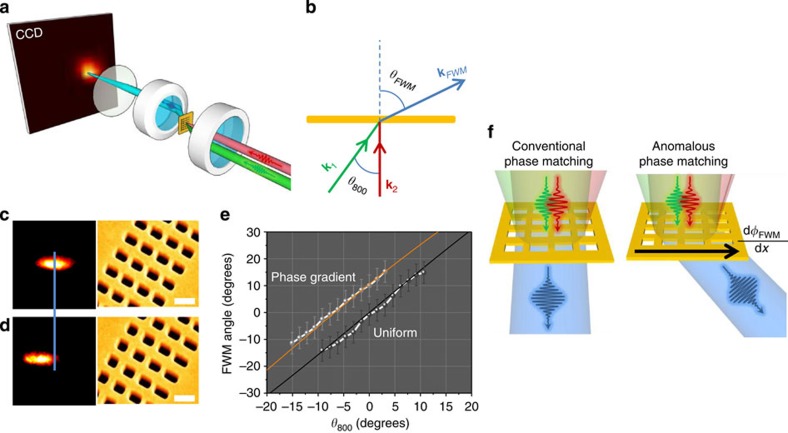
k-Space analysis of the FWM. (**a**) Optical arrangement for measuring the FWM angle dependence. (**b**) The position of the 800 nm (green) beam on the focusing lens, as determined by a translation stage, controls the input angle. The *θ*_FWM_ was determined in relation to the beam generated by a uniform structure. (**c**) CCD image of a signal from a uniform unit cell, and (**d**) from a phase gradient unit cell (the scale bars in the SEM are 500 nm). (**e**) Input angle dependence of the phase-matching angle for the uniform and phase-gradient metasurfaces. The orange line is the line fit to the anomalous phase-matching condition (equation (4)), while the black line depicts the conventional phase-matching condition. (**f**) Illustration of the anomalous phase-matching condition for phase-gradient metasurfaces.

The experimental arrangement is the standard, forward propagating FWM configuration described in an earlier publication^27^. Two 60-fsec beams, one from a Ti:Sapphire and one from an Optical Parametric Amplifier serve as our inputs—their input angles and time delays are individually controllable. In Fig. 2a, the two ultrashort pulses, with wavevectors **k**_1_ and **k**_2_, respectively, are now spatially and temporally overlapped and focused on the phase-gradient metasurface to generate a FWM signal at *ω*_FWM_=633 nm and **k**_FWM_. After the sample, the fundamental beams are filtered and the FWM signal is imaged on a CCD camera which records its k-space information. We measure the FWM from two different metasurfaces—each consisting of four rectangles 450 nm apart, in the uniform case all rectangles are with AR=1.9, and in the phase gradient case the AR covers the range of AR=1.1–2.9.

In both cases we measure the FWM output angle as a function of the input angle *θ*_800_ of the *ω*_1_=800 nm beam for normal incidence of the *ω*_2_ beam. There is an ∼10° difference in the output angle from the two different metasurfaces, indicating a different phase-matching condition. This new phase-matching condition stems from the additional momentum provided by the metasurface, along the gradient direction: 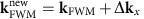, where **k**_FWM_=2**k**_1_−**k**_2_ is the conventional phase-matching condition. The net momentum provided by the metasurface to the FWM signal, Δ**k**_*x*_=(d*Φ*_FWM_/d*x*)**u**_*x*_, is transferred by the metasurface to the beams participating in the nonlinear conversion process.

Thus, the new anomalous phase-matching condition for FWM assumes the form:


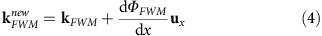


Equation (4) combined with the NL-FDTD calculation for *Φ*_FWM_ provides a framework for the design of phase-gradient nonlinear metasurfaces. In Fig. 2e, the orange curve represents a nonlinear fit to equation (4), from which we extract a value for the phase gradient provided by the metasurface over a unit cell, in this case Δ*Φ*_FWM_=−0.55*π*.

**Figure 3:**
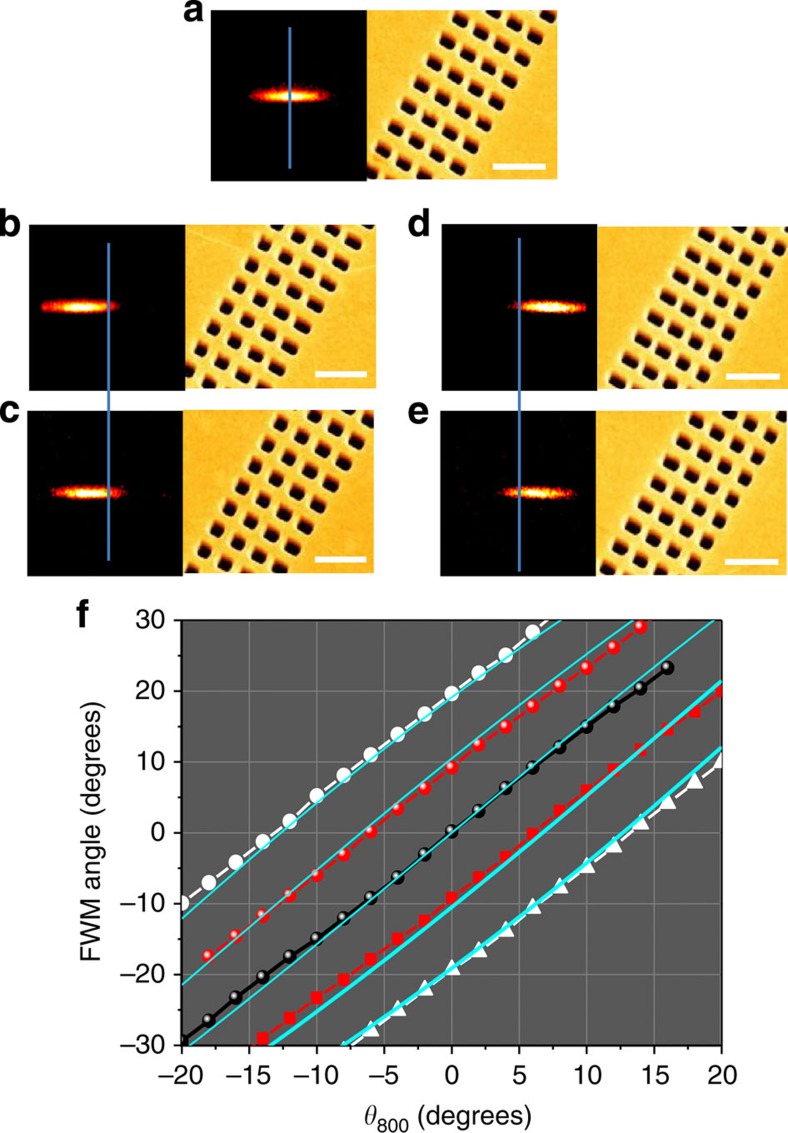
Beam steering angle of the FWM signal for phase-gradient metasurfaces. (**a**–**e**) Experimentally measured phase-gradient structures and the generated FWM at normal incidence. (**a**) Uniform structures; (**b**,**c**) AR increasing to the right; (**d**,**e**) AR increasing to the left. Scale bar, 1 μm (in all s.e.m.'s). (**f**) Calculated FWM angle dependence for phase gradient structures, where the phase gradient was taken from data such as in Fig. 1 and the calculated blue lines are derived from the anomalous phase-matching condition equation (4). The phase gradients d*Φ*_FWM_/*dx* 0 (black circles), −1.1 (white circles), −0.58 (red circles), 0.58 (red squares), and 1.1 (white triangles).

With the proper choice of d*Φ*_FWM_/d*x*, one can control the beam steering of the FWM emission. In Fig. 3, we show a NL-FDTD calculation, using parameters similar to the experimental, of the angle dependence of the FWM signal for different phase-gradient metasurfaces. The line fits to equation (4) using the nonlinear phase gradient shown in Fig. 1 (blue lines) are also plotted and are in good agreement to the NL-FDTD calculations.

**Figure 4:**
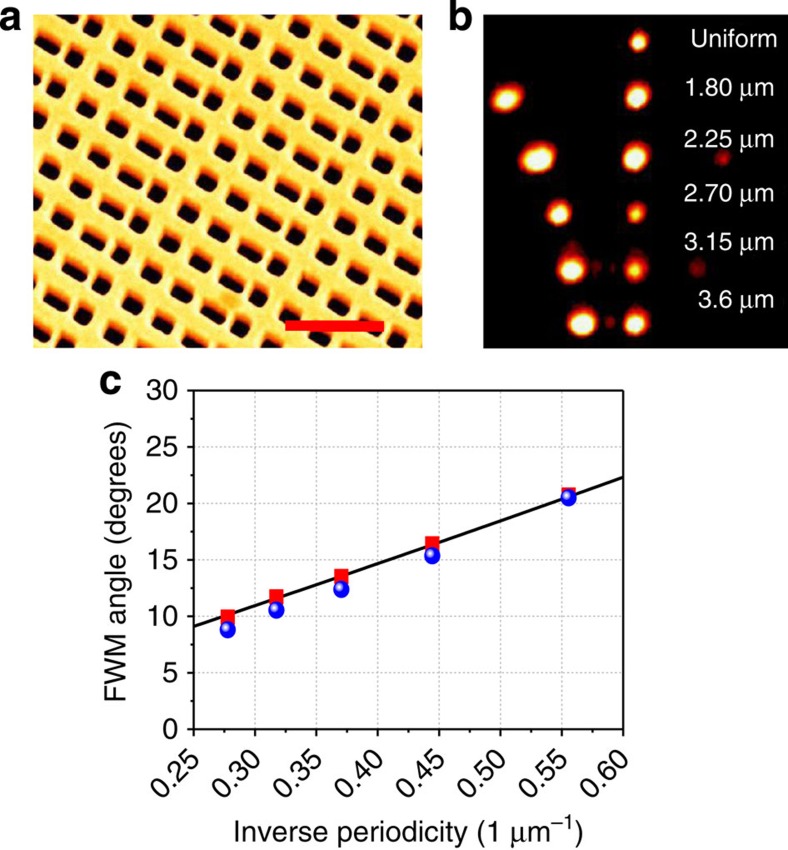
Nonlinear blazed gratings based on periodic arrangement of phase gradient elements. (**a**) Blazed grating with unitary cell consisting of four elements with AR=1.1, 1.5, 1.9, 2.9. Scale bar, 1 μm. (**b**) CCD images for the zeroth and first diffraction orders for different periodicities. A much weaker first negative order may also be seen. (**c**) Measured (blue circles) FWM emission angle as a function of the grating periodicity. The red squares are the results from NL-FDTD simulations and the black line is the prediction of the Raman–Nath NL diffraction theory.

Beyond the phase gradient unit cell, if several (many) cells are arranged in a periodic manner, they form a blazed grating. The analysis of the anomalous phase matching from such a grating should include the general theory of diffraction^24^ in the linear case, or the Raman–Nath diffraction^30^ in the nonlinear one. These blazed gratings, unlike gratings resulting from alternating positive- and negative-phased antennas in uniform unit cells^19^,^31^, are not symmetric in terms of ‘positive and ‘negative' transverse directions, resulting in much lower diffraction efficiency of the negatively diffracted orders (*m*=−1, −2…). In Fig. 4, the scattering from blazed gratings is depicted. The spots seen on the CCD images for collinear, normal excitation are explained by the different orders of diffraction of the blazed grating. The angle of diffraction of the different diffraction orders is determined by the grating period, and agrees with the Raman–Nath diffraction formula sin *θ*_*m*_=*mλ*_FWM_/Λ, where Λ is the period of the grating. High-order diffraction modes are also seen, but with weaker intensity compared with the zeroth and first order, also in accordance with the Raman–Nath diffraction theory.

### FWM metasurface lenses

To further illustrate the power and flexibility of these nonlinear phase-gradient metasurfaces we designed nonlinear metalenses that focus the wavelength of choice in a specific FWM configuration. The ultrathin lenses operate by imposing a radially dependent relative phase shift at the FWM wavelength *ω*_FWM_=2*ω*_1_−*ω*_2_=633 nm:


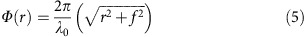


**Figure 5:**
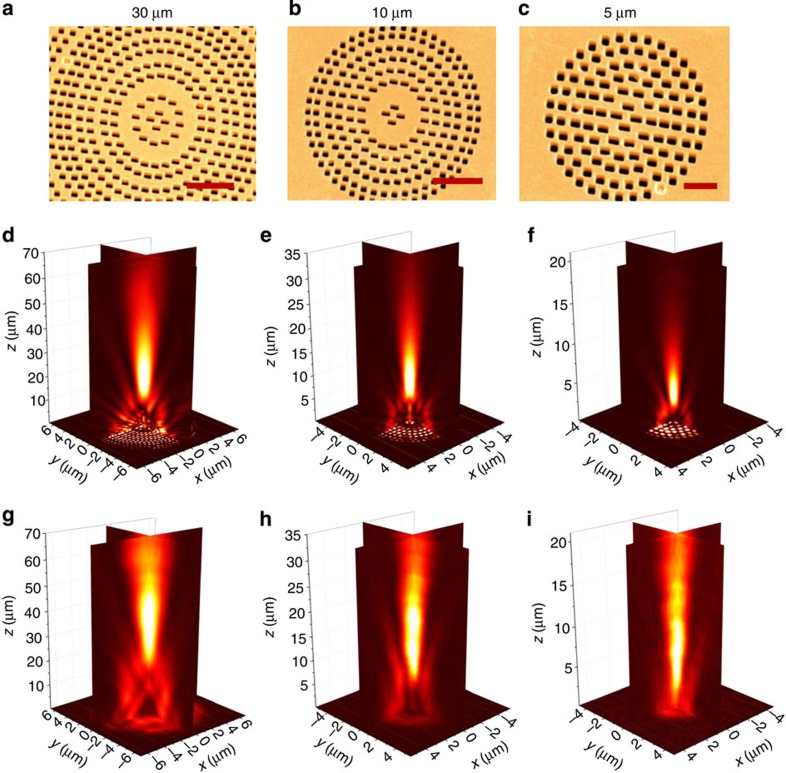
Nonlinear metalenses of focal lengths of 5, 10, 30 μm based on FWM operating at *ω*_FWM_=633 nm. (**a**–**c**) SEM images of the fabricated ultrathin lenses; (**d**–**f**) NL-3D-FDTD simulated images of the focal region; (**g**–**i**) metalens experimental measurement of the focal region. Scale bars, 5 μm (**a**); 2 μm (**b**); 1 μm (**c**).

Here *f* is the desired focal distance of the lens and *λ*_0_ is the free-space wavelength. Metalenses with different focal lengths are shown and discussed in Fig. 5. They are made of concentric rings of phased gratings consisting of rectangular nanoantennas, and the FWM signal is a tightly focused, nearly diffraction limited Gaussian spot. Note that the experimentally observed focal spots are not as tight as the calculated ones. This discrepancy may be attributed to less than perfect fabrication accuracy, and input beams which are not fully collimated. Both factors will give rise to different focal parameters.

## Discussion

In the present work, we demonstrate full control over the nonlinear phase on the subwavelength scale in phase-gradient metasurfaces. As in the linear regime, where Snell's law had to be modified, the phase control over the nonlinear nanoantennas leads to a modified manifestation of the laws governing nonlinear phenomena, such as NL scattering, NL refraction and frequency conversion. Related phase control had been previously reported for the linear case and analysed in terms of the Berry phase^9^, and more recently for nonlinear harmonic generation^19^,^20^, while others have discussed shape resonances and their effect on SHG^17^,^32^,^33^. For a recent review of nonlinear plasmonics see ref. 11 and references therein. The present implementation of the subwavelength phase gradients enables treatment of any polarizations and for any input frequency.

Quite a few ultrathin optical components^6^,^7^,^34^,^35^,^36^ have been proposed and used for beam steering applications. These are based on the abrupt phase changes experienced by light on propagation through a properly designed ultrathin layer of metamaterial. The beam bending (at a specific angle) results from scattering by phase gratings generated by the proper design of the nanoantennas. These nanoantennas are generally uniform across a unit cell, and their phase changes abruptly (typically by *π*) at a predesigned periodicity. We have carried this concept one step further, designed and fabricated blazed metasurfaces, where the scattering is from the phase gradient across the unit cell, and carefully analysed their nonlinear optical properties. We show that FWM from such metasurfaces reveals a new feature: the scattering from a phase gradient unit cell enables anomalous phase-matching condition for FWM from such metasurfaces. This phase-matched FWM is efficient; its phase-matched direction of propagation may be controlled by proper design of the phase gradients, it enables beam bending at any angle and thus FWM focusing, and it differs from the scattering by phase gratings, as discussed in ref. 24. In all these cases, the experimental results were compared with numerical solutions (NL-FDTD, Lumerical solutions package^37^) of the wave equations with nonlinear terms added to them. The agreement is generally very good, and whenever relevant, comparison with analytical expressions is added.

We designed and fabricated ultrathin FWM lenses, and demonstrated tight focusing with focal lengths of several microns. These FWM lenses do not have any restrictions on the symmetry of the design which is characteristic to elements based on SHG, and can be integrated in light detectors based on frequency conversion to provide more sensitive detection.

For pedagogical reasons we have limited our design to rectangles and the tuning parameter to the AR, but more generally parameters such as area of the hole, or shapes presenting multiple resonances such as V-shaped antennas can and will be used. Interestingly, even though the linear rectangular nanoatennas do not cover the full range of 2*π* phase shift, it is possible to obtain a 2*π* phase shift in the nonlinear case due to multiples or combinations of linear phases imprinted on the fundamental beams.

As is well known, metal structures are lossy, especially in the visible range, and thus the search is going on for nonmetallic replacements. This endeavour, however, is hampered by the lack of the electromagnetic enhancement readily available for metals, and presently combined structures consisting of nonlinear materials with plasmonic metallic structures seem to offer a pathway towards higher efficiencies^21^.

In conclusion, we demonstrate full control over the nonlinear phase in phase-gradient metasurfaces. We show that in such metasurfaces a new phase-matching condition applies, which differs from the phase-matching schemes known in nonlinear optics, and have designed and built ultrathin elements such as blazed elements and lenses that are based on nonlinear wave mixing. The phase control of nonlinear nanoantennas will enable the design of ultrathin nonlinear metamaterials, which can generate and control the wavefront of light, and may have implications for the next generation of efficient devices for spectroscopic (CARS or SERS) measurements.

## Methods

### Sample fabrication

The samples were fabricated by focused ion beam (FIB) milling on a high quality free-standing gold film. The procedure for fabrication of free-standing gold films is described in details in ref. 27 Briefly, using an e-beam evaporator, we deposited a 10-nm thick Cr adhesion layer and a 250-nm thick gold layer on a polished silicon wafer. On the opposite side of the wafer, we had previously grown a circular Si_3_N_4_ mask by plasma-enhanced chemical vapour deposition. The mask was chemically etched using KOH and the remaining free-standing metallic area was etched with HCl to remove the adhesion layer.

### Linear FDTD simulations

The transmittance spectrum and the relative spectral phase response of the rectangular metallic nanocavities were calculated using the commercial software Lumerical FDTD solutions. The values of the dielectric constants were taken from the data table of Gold from Palik^38^.

### Nonlinear FDTD simulations

The nonlinear phase was calculated using the nonlinear material implementation of Lumerical. The base material is Palik gold, which is assumed to have instantaneous (non-dispersive) third-order nonlinearity *χ*^(3)^=10^−18^ m^2^ V^−2^. As input light sources we used two temporally overlapped transform-limited plane wave sources centred at *ω*_1_=800 nm and *ω*_2_=1,088 nm, with pulse duration 60 fs, propagating parallel to the *z* direction. The polarization of both pulses is perpendicular to the long axis of the rectangles. The *y* component of the real and imaginary parts of the electric field (that is, the propagating waves) of the FWM signal is recorded on a *y* normal plane spanning the whole simulation area. The dimensions of the mesh were set to d*x*=d*y*=d*z*=5 nm and perfectly matched layers were added in all dimensions.

### k-Space analysis

For the k-space analysis of the FWM signal from phase-gradient antennas, the same simulation parameters used in the phase response were kept, except now the *ω*_1_=800 nm source propagates in the *x*–*z* plane with a variable incidence angle *θ*_800_ with respect to the normal. The exit fields on the opposite side of the metasurface are recorded in a *z*-normal plane and projected to the far field, where the angle *θ*_FWM_ of the FWM signal at *ω*_FWM_=633 nm is calculated as a function of *θ*_800_.

### K-space measurements

In the FWM experiments, we used the set-up described in details in ref. 27 An optical parametric amplifier, pumped by a 1-kHz amplified Ti:Sapphire laser, was used as the light source for the *ω*_2_=1,088 nm pulses, while the pulses of the Ti:Sapphire laser that pumped the optical parametric amplifier were used as the fundamental *ω*_1_=800 nm beam. Both *ω*_1_ and *ω*_2_ pulses have the same pulse duration of 60 fs. The beams travel two distinct optical paths, where the intensity and polarization of each individual beam could be controlled by a set of half-wave plate and polarizer to avoid optical damage to the samples. Both beams are focused and overlapped in the sample, by an objective lens of numerical aperture (N.A.)=0.42 (Mitutoyo M Plan Apo 50X Infinity-Corrected). The incident angle *θ*_800_ of the *ω*_1_ beam can be varied by controlling the lateral displacement of the beam with computer-controlled translation stage supporting a beam splitter whose primary role was to merge the optical path of two beams. The *ω*_2_ beam at 1,088 nm was always kept normal to the surface. The temporal overlap between the two beams as the input angle was varied, was monitored and controlled by properly delaying the *ω*_1_ beam. The FWM signal centred at *ω*_FWM_=633 nm is collected by an objective lens with N.A.=0.42 (Mitutoyo M Plan Apo SL50X Infinity-Corrected) and focused by spherical lens of *f*=75 mm onto an EMCCD camera (Andor iXon DV885) The fundamental beams are filtered by a pair of shortpass filters (Thorlabs FES0700 and FESH0750). In the angular measurements in Figs 3 and 4, the focusing objective with N.A.=0.42 was replaced by a lens of focal length *f*=50 mm to illuminate a larger area.

### Nonlinear lenses

For the design of our lenses we kept the same parameters used in the calculations of the NL phase response. However, to decrease the simulation time, we used symmetric boundary condition in the *x* dimension and anti-symmetric in the *y* dimension. The dimensions of the fine mesh around the lenses were set to d*x*=d*y*=10 nm and d*z*=5 nm for the *f*=5 and 10 μm lenses and d*x*=d*y*=15 nm and d*z*=5 nm for the *f*=30 and 60 μm lenses.

### Nonlinear lenses measurements

The experimental set-up for measurements of the nonlinear lenses is a modification of the k-space set-up described above. Both *ω*_1_ and *ω*_2_ beams are normally incident. The focusing objective is replaced by a spherical lens with *f*=100 mm. The FWM signal out of the lenses is collected by the imaging (NA=0.42) objective and is imaged directly onto the EMCCD in real (physical) space. The imaging objective is supported on a computer-controlled translation stage that can vary the focal plane of the objective and record three-dimensional tomographic images of the focal region.

## Additional information

**How to cite this article**: Almeida, E. *et al*. Subwavelength nonlinear phase control and anomalous phase matching in plasmonic metasurfaces. *Nat. Commun.* 7:10367 doi: 10.1038/ncomms10367 (2016).
